# The emerging role of the piRNA/piwi complex in cancer

**DOI:** 10.1186/s12943-019-1052-9

**Published:** 2019-08-09

**Authors:** Yongmei Liu, Mei Dou, Xuxia Song, Yanhan Dong, Si Liu, Haoran Liu, Jiaping Tao, Wenjing Li, Xunhua Yin, Wenhua Xu

**Affiliations:** 1 Department of Inspection, The medical faculty of Qingdao University, Qingdao, 266003 China; 2 School of Public Health, Qingdao University, Qingdao, 266003 China; 3 The Laboratory of Biomedical Center, Qingdao University, Qingdao, 266003 China; 4 Institute of Translational Medicine, Qingdao University, Qingdao, 266003 China

**Keywords:** piRNA/piwi complex, Cancer, Function, Biomarker

## Abstract

Piwi interacting RNAs (piRNAs) constitute novel small non-coding RNA molecules of approximately 24–31 nucleotides in length that often bind to members of the piwi protein family to play regulatory roles. Recently, emerging evidence suggests that in addition to the mammalian germline, piRNAs are also expressed in a tissue-specific manner in a variety of human tissues and modulate key signaling pathways at the transcriptional or post-transcriptional level. In addition, a growing number of studies have shown that piRNA and PIWI proteins, which are abnormally expressed in various cancers, may serve as novel biomarkers and therapeutic targets for tumor diagnostics and treatment. However, the functions of piRNAs in cancer and their underlying mechanisms remain incompletely understood. In this review, we discuss current findings regarding piRNA biogenetic processes, functions, and emerging roles in cancer, providing new insights regarding the potential applications of piRNAs and piwi proteins in cancer diagnosis and clinical treatment.

## Background

PIWI-interacting RNAs (piRNAs) constitute a class of recently discovered small non-coding RNAs in germ- and somatic cells comprising 24–31 nucleotides (nt) with a 5′-terminal uridine or tenth position adenosine bias, lacking clear secondary structure motifs [1]. They were first described in 2001 in Drosophila testes as small RNAs derived from the Su(Ste) tandem repeats, which silence Stellate transcripts to maintain male fertility [2]. Unlike miRNAs and siRNAs, which typically rely on RNase type III enzymes to convert double-stranded RNA precursors into functional small RNAs, mature piRNAs derive from an initial transcript encompassing a piRNA cluster via a unique biosynthesis process [3]. piRNAs can bind to piwi proteins to form a piRNA/piwi complex, thereby influencing transposon silencing, spermiogenesis, genome rearrangement, epigenetic regulation, protein regulation, and germ stem-cell maintenance [4]. The piwi family exhibits highly conserved structure and function across multiple organisms [5], including fruit fly (PIWI, Aubergine, and AGO3 proteins) [6], mouse (MILI, MIWI, and MIWI2) [7–11], human (HILI, HIWI1, HIWI2 and HIWIL3) [6, 12–14], zebrafish (ZILI and ZIWI) [15], and nematode (PRG-1 and PRG-2) [16]. Moreover, aberrant piRNA or PIWI protein expression has recently been reported in some human cancer, with some piRNAs/piwi complexes participating in tumorigenesis and associated with cancer prognosis [17–19].

Cancer accounts for 1.2 million deaths annually in China, with an additional 1.6 million people diagnosed each year [20]. Treatments are often ineffective owing to relatively late disease detection combined with high rates of metastasis and recurrence [21], highlighting the need for novel biomarkers of cancer diagnosis and prognosis along with new targets for effective therapeutic approaches. Notably, numerous studies have implicated the piRNAs/piwi complex in the occurrence, development, metastasis, and recurrence of e.g., breast (BC) [22], lung (LC) cancer [17]. The present review summarizes the latest research regarding piRNAs including their biosynthesis, functions, and mechanism, along with their roles in different cancers and as potential biomarkers.

### piRNA biosynthesis mechanism

#### Transcription of piRNA clusters

**Fig. 1:**
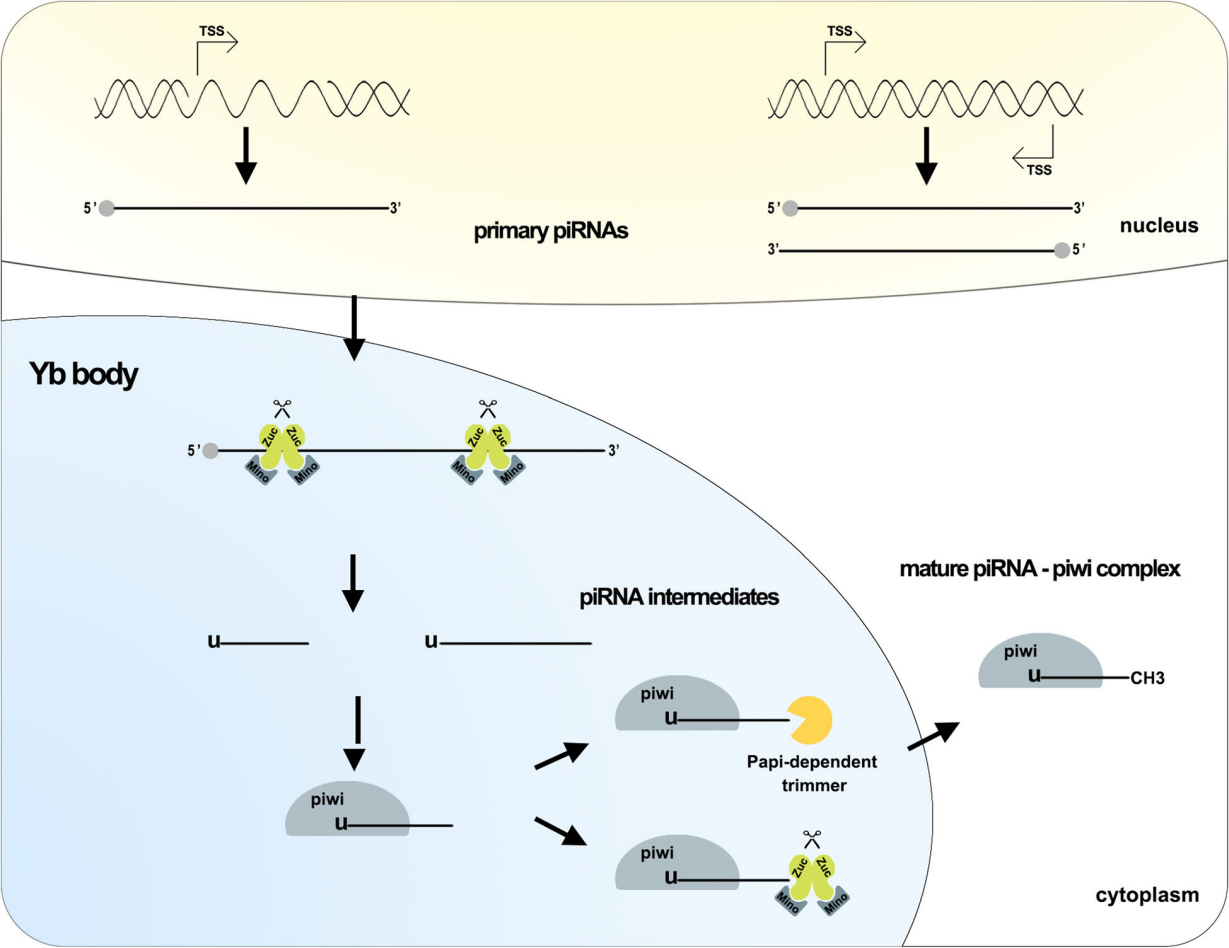
piRNA biosynthesis mechanism. Within the nucleus, two types of piRNA clusters are transcribed to produce the primary piRNAs, Zuc and its co-factors incised primary piRNAs producing piRNA intermediates with a 5′ uracil in Yb body. piRNA intermediates linked piwi that are cleavage by Zuc or Papi-dependent trimmer to form 3’end . Following methylation in cytoplasm, the mature piRNA-piwi complex is producted. Abbreviations: TSS: transcription start site; Zuc: zucchini

A large fraction of piRNAs that can be uniquely mapped originate from two types of extended (up to 200 kb) genomic loci, termed piRNA clusters [23]. Similar to coding genes, uni-stranded clusters contain promoters marked by Pol II Ser5P and H3K4me2 peaks that produce transcripts via RNA polymerase II, which undergo5-terminal capping, 3-terminal polyadenylation, and sometimes selective splicing. Conversely, dual-strand clusters are transcribed from both genomic strands, depend on promoters of nearby coding genes to initiate transcription and are not equivalently processed [3, 23]. (Fig. 1).

#### Two major pathways generate piRNAs

piRNA clusters produce primary piRNAs that are transported to the cytoplasmic Yb body [24, 25]. Zucchini (Zuc) and its co-factor Minotaur (Mino) incise primary piRNAs, producing piRNA intermediates with a 5′ uracil [26, 27]. Piwi protein contains an evolutionarily conserved structure consisting of a PAZ and piwi domain. PAZ preferentially binds piRNA intermediates with 5′ uracil, as observed for silkworm piwi in vitro [28]. Upon binding to piwi protein, piRNAs mature through 3′-end cleavage by the Zuc riboendonuclease [29], or via the Papi-dependent trimmer. Subsequent methylation by Hen1 yields the mature piRNA-piwi complex [3, 30] (Fig. 1).

### piRNA/piwi protein function and mechanism in cancer

**Fig. 2:**
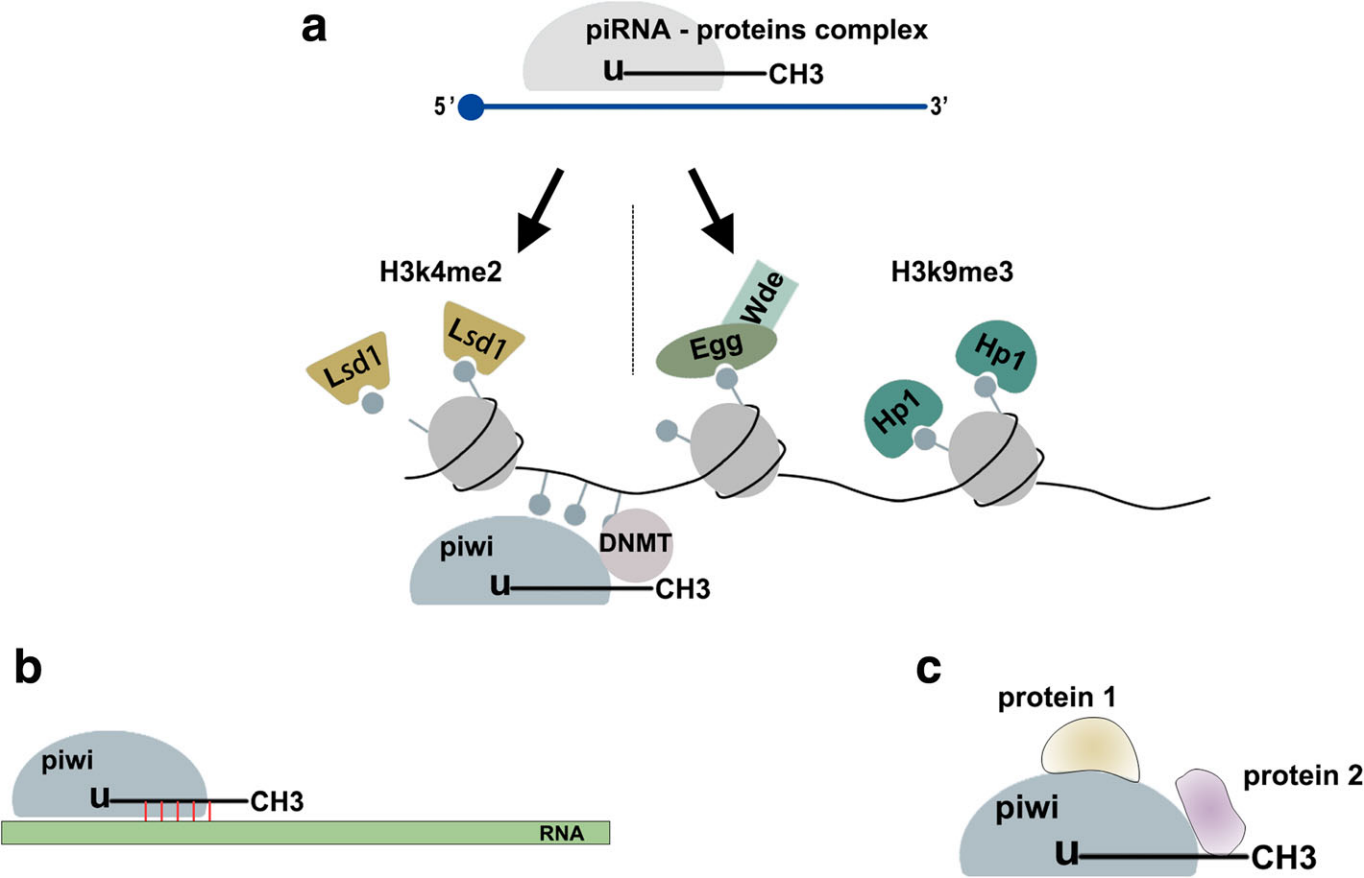
piRNA/piwi protein function. **a**. At TGS level, the piRNA-proteins complex recruit silencing machinery components to bring repressive H3K9me3 marks to target DNA body and remove active H3K4me2 marks from promoter regions. In addition, piRNAs/piwi complex recruits DNMT, results in methylation at CpG sites in genic. **b**. At PTGS level, the piRNAs/piwi complex bind to targeted RNAs and impede their function by sequence complementary. **c**. piRNAs/piwi complex-protein interaction. The interaction between piRNAs/piwi and proteins alter the subcellular localization of proteins and facilitate the interaction of multiple proteins. Abbreviations: TGS: transcription gene silencing; PTGS: post-transcription gene silencing; H3K9me3: histone 3 lysine 9 trimethylation; H3K4me2: histone 3 lysine 4 dimethylation; DNMT: DNA methyltransferase

Recent studies indicate that piRNAs play a vital role in physiological and pathological processes at the transcriptional or post-transcriptional level. Here, we summarize the function and mechanisms of piRNAs in cancer (Fig. 2).

#### piRNAs/piwi complex-mediated transcriptional gene silencing (TGS)

piRNA/piwi complexes enter the nucleus and bind its genomic target through a nascent transcript by sequence complementary. Once combined with Panoramix (Panx), the piRNA-proteins complex induces TGS by recruiting silencing machinery components. Eggless (Egg) and its co-factor Windei (Wde) add repressive histone 3 lysine 9 trimethylation (H3K9me3) marks to the target DNA; subsequently, heterochromatin protein 1 (HP1) is recruited, causing heterochromatin formation. Lysine-specificdemethylase1 (Lsd1) removes activating H3K4me2 marks from promoter regions, inhibiting RNA Pol II transcription [31]. piRNA/piwi complex also recruits DNA methyltransferase (DNMT) to methylate genic CpG sites (non-transposable element (TE) protein-coding), altering transcriptional activity [32](Fig. 2a). In Aplysia neurons, an endogenously expressed piRNA induced CREB2 promoter methylation [33]. Furthermore, expression of a “piRNA-like” 28-nt transcript antisense to the KIR3DL1 promoter strongly correlated with KIR3DL1 promoter methylation in CD56^+^ natural killer cells [34]. piR-021285 overexpression facilitated ARHGAP11A methylation at a CpG site within the 5′UTR/first exon, decreasing mRNA (pro-apoptosis) expression and inhibiting BC cell apoptosis [35]. In multiple myeloma (MM), piRNA-823 directly recruited de novo DNA methyltransferases DNMT3A and DNMT3B in primary CD138^+^ MM cells, increasing global DNA methylation and inhibiting tumor suppressor p16_INK4A_ expression [36].

#### piRNAs/piwi complex- mediated post-transcriptional gene silencing (PTGS)

Numerous studies have found that many piRNAs regulate post-transcriptional networks to inhibit target function through piRNA-RNA interactions, similar to miRNA mechanisms. These RNAs include mRNA [37], transcribed pseudogenes [22], and long noncoding RNA (lncRNA) [38]. Effective mRNA: piRNA interaction requires strict base pairing within 2–11 nt at the 5′-end of piRNA and less stringent base pairing within 12–21 nt [39] (Fig. 2b). Functional piRNA-induced silencing complexes (pi-RISCs), comprised of MIWI, piRNAs, and CAF1 deadenylase in mouse elongating spermatids, mediate mRNA deadenylation and decay via an miRNA-like mechanism with guider piRNAs and CAF1. The resulting elimination of large mRNA quantities may promote nucleus condensation and cytoplasm exclusion to complete spermatozoa formation in mammals [40]. piRNA-piwi complexes also recruit carbon catabolite-repressed 4-negative on TATA-less (CCR4–NOT) and Smaug (Smg) to form specific pi-RISCs, which promote RNA repression through imperfect base-pairing via an miRNA-like mechanism [40–42]. piR-55490 binds the mTOR 3′-UTR, causing mRNA degradation and suppression of LC development [37].

piR-30188 binds the lncRNA OIP5-AS1 and inhibits OIP5-AS1 expression, thereby suppressing glioma cell malignant phenotype via the miR-367/CEBPA/TRAF4 pathway [38]. The piRNA-piwi ribonucleoprotein complex also maintains genome integrity by post-transcriptionally silencing TEs [41], which can drive genome evolution and must be tightly regulated as their over-activity is detrimental to the host [5]. In Ping-Pong piRNA amplification, symmetric dimethyl-arginine (sDMA)-modified mature ribonucleoprotein complexes are recruited by Krimper, which also interacts with unloaded Ago3, thus bringing these together [3]. As both contain piwi domains with RNase H endonuclease activity [43]. The compound can selectively detect and slice transposon RNAs to post-transcriptionally silence TEs, maintaining genome integrity [3].

#### piRNAs/piwi complex interaction with proteins

The piRNAs/piwi complex directly binds to some proteins by piRNAs or the piwi protein PAZ domain, as shown by piRNAs/piwi complex and target protein co-localization. Such interaction facilitates multi-protein interactions, altering their subcellular localization (Fig. 2c). piR-823 interacts with heat shock factor 1(HSF1) to promote Ser326 phosphorylation and HSF1 activation, thereby enhancing colorectal cancer (CRC) cell proliferation and suppressing cell apoptosis [44]. piR-54265/PIWIL2 recruits STAT3 and p-SRC to form the PIWIL2/STAT3/p-SRC complex via the PIWIL2 PAZ domain, facilitating p-SRC-mediated STAT3 phosphorylation and signal pathway activation to promote tumorigenesis [45].

### piRNAs in cancer

Numerous piRNAs are dysregulated in tumor tissues, playing tumor-promoting or tumor-suppressor roles. Growing evidence shows that piRNAs strongly correlate with tumor cell malignant phenotype and clinical stage. Here, we summarize recent studies regarding mechanisms of piRNAs in various cancers (Table 1).

**Table 1:** The Role of piRNAs in various Cancer

piRNA	Cancer type	Function	Expression in tumors	Reference
piR-36712	Breast cancer	suppressed cell proliferation, invasion and migration by combining with SEPW1P RNA	down	[22]
piR-021285		inhibited cell proliferation and invasion by ARHGAP11A methylation	down	[35]
piR-932		caused EMT through promoting promoter region CpG island methylation of Latexin	up	[46]
piR-DQ598677		form pi-RISC to degrade targeted genes like miRNAs	down	[47]
piR-34871 piR-52200	Lung cancer	correlated with RASSF1C expression, promoted cell proliferation and colony formation by reducing AMPK phosphorylation of ATM-AMPK-p53-p21cip pathway	up	[17]
piR-35127 piR-46545			down	
piR-651		Promoted cells and tumor proliferation and inhibited apoptosis, induced cyclin D1 and CDK4 expression	up	[48, 49]
piR-55490		inhibited LC cells and tumor proliferation by binding 3’UTR of mTOR mRNA	down	[37]
piR-823	Gastric cancer	inhibited proliferation of cancer cells, and caused cells aberrant “stem-like”state by weakening tumor supporter genes methylation	down	[19, 50]
piR-651		promote cell proliferation and associated with TNM stages	up	[51]
piR- FR222326		positively associated with overall survival	up	[52]
piR-FR290353 piR-FR064000 piR-FR387750		associated with recurrence-free survival	up	[52]
piR-1245	Colorectal cancer	accelerated cell growth, promoted migration and invasion as well as anti-apoptosis by binding to its downstream targeted mRNA in nuclear exosomes, associated with poor differentiation, TNM state and poor overall survival	up	[53]
piR-54265		promoted proliferation and metastasis, inhibited apoptosis, correlated with shorter progression-free survival time and overall survival time, caused therapy resistance to anti-tumor agents by regulating STAT3 phosphorylation	up	[45]
piR-823		enhanced cells proliferation and suppressed apoptosis by promoting HSF1 phosphorylation at Ser326 and inducing Stat3 phosphorylation	up	[44]
piR-015551		influenced the colorectal cancer development by causing gene mutation	up	[54]
piR-Hep1	Hepatocellular carcinoma	promoted cells proliferation and invasion via upregulating phosphorylated AKT of PI3K/AKT signaling pathway	up	[55]
piR-LLi-24894		asssociated with low-grade lesions of hepatocellular carcinoma	up	[56]
Hsa-piR-013306		involved in the hepatic carcinogenic process	up	[56]
piR-32051 piR-39894 piR-43607	Kidney cancer	linked with renal cell carcinoma of high tumor stage and metastasis and cancer-specific survival	up	[57]
piR-57125		inhibited cancer metastatic	down	[58]
piR-30924 piR-38756		associated with cancer metastatic	up/down	[58]
piR-823	Hematological malignancy	promoted proliferation, inhibited apoptosis and modulated cell cycle progression of multiple myeloma cells by regulating DNA methylation and angiogenesis	up	[36]
		improved the survival and maintained the stemness of multiple myeloma stem cells by producing more DNMT3B	up	[59]
		promoted the proliferation, migration, and capillary structure formation of tumor-associated endothelial cells	up	[60]
piR-651		associated with shorter disease-free survival and shorter overall survival in classical Hodgkin lymphoma patients	up	[61]
piR-30188	Glioblastoma	supressed tumor cell proliferation, invasion and migration and promoted apoptosis by binding to OIP5-AS1	down	[38]
piR-8041		Promoted cells proliferation and inhibited death by interacting with the mRNA MAPK	down	[62]
piR-DQ593109		increased the permeability of the blood-tumor barrier and promoted the delivery of therapeutics into the glioma micro-environment via bindind to MEG3	down	[63]
piR-DQ590027		increased the permeability of glioma-conditioned normal blood-brain barrier and promoted the transport of macromolecular chemotherapeutics into glioma tissues by binding to MIR17HG	down	[64]
piR-39980	Fibrosarcoma	inhibition of cell proliferation via interacting with RRM2	down	[65]
piR-52207	Ovarian cancer	promoted cell proliferation, migration and tumorigenesis by binding to targeted mRNA (NUDT4, MTR, EIF2S3, MPHOSPH8)	up	[66]
piR-33733		Inhibited cells apoptosis by binding to targeted mRNA (ACTR10, PLEKHA5)	up	[66]
piR-017061	Pancreatic cancer	Not clear	down	[67]

#### Breast cancer (BC)

BC constitutes the most commonly diagnosed cancer (25%) and major cause of cancer death (15%) among women worldwide [68]. The piR-36712/PIWIL1 complex, which suppresses cell proliferation, invasion, and migration through the piR-36712/SEPW1P RNA/miR-7/− 324/P53/P21 axis, negatively correlates with tumor size and metastases. SEPWEP1competes as a competitive endogenous RNA (ceRNA) with SEPW1 RNA for miR-7 and miR-324. piR-36712 over-expression-mediated SEPWE1P recruitment decreases SEPW1 expression and enhances P53 and P21 activities by inhibiting their ubiquitin-mediated degradation, resulting in G1 cell cycle arrest. piRNA-36,712 also shows synergistic anticancer effects with the BC chemotherapeutic agents paclitaxel and doxorubicin. AgopiR-36,712 treatment inhibits growth of MCF7 or ZR75–1 cell-derived xenografts in vivo. Thus, piR-36712 represents a tumor suppressive non-coding RNA and a therapeutic target in BC [22]. piR-021285 regulates cell proliferation and invasion by DNA methylation. Variant piR-021285-mimic transfection into BC cell lines weakens ARHGAP11A pro-invasive and pro-apoptosis gene methylation at a 5′-UTR/first exon CpG site, resulting in higher ARHGAP11A expression and increased BC cell invasiveness [35]. Similarly, piR-932 and PIWIL2, which constitutes a bridge between cancer stem cells (CSCs) and proliferation and anti-apoptosis, form a complex to promote latexin promoter CpG island methylation in BC stem cells [46]. Latexin, a tumor suppressor, reduces old stem cell transformation into CSCs, decreases cell replication, and increases apoptosis [69, 70]. Increased piR-932/PIWIL2 complexes reduces latexin expression, promoting epithelial-mesenchymal transition (EMT) in BC [46]. piR-DQ598677, which is down-regulated in BC, inhibits BC growth post-transcriptionally through piRNA-RNA imperfect base-pairing-mediated RNA degradation as it is complementary to the 5′-UTR, 3′-UTR, and coding region of TAX1BP, TNFESF10B, and SFRP2 mRNA, respectively, which are involved in key cancer cell functions such as cell-to-cell signaling and interaction, cell death and survival, and cell cycle [47].

#### Lung cancer(LC)

LC has the highest incidence and mortality among all cancers, with a low 5-year survival rate [71]. The tumor promoter RASSF1C up-regulates piR-34871 and piR-52200 and down-regulates piR-35127 and piR-46545 through the RASSF1C-PIWIL1-piRNA axis to promoted LC stem cell proliferation, colony formation, and EMT. These piRNA changes inhibit AMPK phosphorylation in the ATM-AMPK-p53-p21_cip_ pathway, resulting in lung cell EMT and enhancing epidermal growth factor receptor (EGFR) signaling, blocking cell cycle arrest and enhancing cell proliferation [17]. piR-651 regulates tumorigenesis in 95-D high metastasis human LC cells by inhibiting apoptosis and altering apoptosis-related protein expression. piR-651 inhibitor treatment enhances apoptosis-related protein expression including caspase3 and bax, consequently restraining tumor progression [48]. Furthermore, up-regulated piR-651 in non-small cell lung carcinoma (NSCLC) may induce oncogene expression, such as cyclin D1 and cyclin-dependent kinase 4 (CDK4), although the exact mechanism remains unclear. Up-regulated cyclin D1 and CDK4 promotes cell cycle progression, resulting in cell proliferation [49]. piR-55490 binding to the 3′-UTR of mTOR inhibits the expression of mTOR and its target genes, HIF-1, PGC-1α, and PPARγ, reducing LC cell and tumor proliferation [37], as the Akt/mTOR signaling pathway is a key cancer biology pathway [72]. Furthermore, piR-55490 expression negatively correlates with patient survival. Notably, Ad-piR-55490 treatment suppresses LC cell proliferation, supporting piR-55490 as a therapeutic target [37].

#### Gastric carcinoma (GC)

GC incidence is 2 fold higher in men than women but varies between countries [73]. piRNAs that mediate transposon silencing during normal germline differentiation can be hijacked in cancer cells to silence other parts of the genome, resulting in tumorigenesis [74]. Alternatively, piR-923 down-regulation in GC tissue correlates with cancer cell proliferation and contributes to the precancerous stage of stomach carcinogenesis. Abnormally expressed piRNAs may also cause aberrant DNA methylation and activate genomic regions (potentially including tumor promoting genes), producing an aberrantly “stem-like” state and consequent tumorigenesis. piR-823 mimic treatment suppressed tumor growth and GC cell proliferation in vivo and in vitro, suggesting piR-823 as an attractive therapeutic target for GC [19, 50]. piR-651 is up-regulated in e.g., human GC, BC, LC, and hepatic carcinoma cell lines, indicating that piR-651 may function as a critical oncogene in carcinogenesis. piR-651 promotes GC cells to enter the G2/M phase to promote cell proliferation. piR-651 levels are also associated with TNM stages, with low differentiated cancers associated with elevated piR-651. Transfecting a piR-651 inhibitor into GC cells dose-dependently inhibited cell growth, suggesting piR-651 as a potential target for cancer therapy [51]. Most piRNAs associated with gastric adenocarcinoma are embedded in protein-coding sequences rather than known piRNA clusters from the gastric piRNAs atlas. Only one piRNA, FR222326, positively associated with overall survival (OS). A three-piRNA cluster (FR290353, FR064000, FR387750/FR157678) associated with recurrence-free survival (RFS) effectively stratified patients with gastric adenocarcinoma into low-risk and high-risk recurrence groups. However, further research is required to clarify the specific mechanisms [52].

#### Colorectal cancer (CRC)

CRC is the third frequent cancer in men and the second in women [73]. High piR-1245 expression accelerates CRC cell growth, promotes migration and invasion, and inhibits apoptosis. piR-1245 binds through sequence complementarity, to the intronic regions of its targeted mRNAs (ATF3, BTG1, DUSP1, FAS, NFKBIA, UPP1, SESN2, TP53INP1, and MDX1), which are involved in key tumor suppressive pathways, promoting mRNA degradation via nuclear exosomes. High piR-1245 expression is significantly more pronounced in CRC tissues with poor differentiation, advanced T stage, lymph node metastasis, distant metastasis, and poor OS [53]. piR-54265 is also increased in CRC tumor tissue, and promotes CRC cell proliferation and metastasis, and inhibits apoptosis through PIWIL2/STAT3/p-SRC complex formation, in which STAT3 is phosphorylatively activated by p-SRC, and subsequent anti-apoptotic BCL-XL and pro-metastatic matrix metalloproteinase-2 (MMP2) and MMP9 up-regulation. High piR-54265 levels correlate with shorter progression-free survival (PFS) and OS. In addition, piR-54265 over-expression increases 5-FU and oxaliplatin half maximal inhibitory concentrations (IC50), causing chemoresistance. Notably, however, piRNA-54,265 inhibitor treatment significantly suppressed implanted tumor growth and metastasis [45]. piR-823 is also up-regulated in CRC tissues and enhances CRC cell proliferation and suppresses cell apoptosis by heat shock factor 1 (HSF1) at a post-translational level. Specifically, piR-823 interacts with HSF1, a common transcription factor that can regulate heat shock proteins (HSPs) expression, to promote its phosphorylation at Ser326, inducing HSF1 activation [44]. HSF1, highly expressed various cancers, is a strong driver of carcinogenesis including CRC [44, 75–77]. CRC progression may also occur via piR-823/piwil2 complex-mediated STAT3 phosphorylation and STAT3/BCL-xl/cyclinD1 signaling pathway activation, which can induce CDK inhibitor (CDKI) expression and regulate G1 phase progression. piR-823 inhibitor treatment induces G1 phase stagnation and decreases G1 phase regulator cyclin D1 and CDK4 expression, consequently inhibiting CRC cell proliferation and promoting cell apoptosis, supporting piR-823 as a therapeutic target [44]. LNC00964–3 includes sequence for piR-015551, which shows elevated expression in CRC tissues. Moreover, the piR-015551 rs11776042 variant (thymine to cytosine; T > C) modifies piRNA secondary structure, which influences piRNA effects on CRC development [54].

#### Hepatocellular carcinoma (HCC)

Liver cancer constitutes the second and sixth leading cause of cancer mortality among men in developing and developed countries, respectively [68]. Up-regulated piR-Hep1 in HCC promotes hepatocellular proliferation and invasion, potentially by binding with PIWIL2 to up-regulate phosphorylated AKT in thePI3K/AKT signaling pathway [55], a key oncogenic pathway in HCC [78]. High piR_LLi_24,894 indicates low-grade HCC lesions; moreover, significant hsa_piR_013306 accumulation only presents in HCC, suggesting the direct or indirect involvement of piRNAs in the hepatic carcinogenic process [56].

#### Kidney cancer(KC)

KC is difficult to detect and treat, and is poorly understood [79]. Renal cell cancer (RCC) accounts for 2.4% of all adult malignancies worldwide with continuously increasing incidence and high cancer-specific mortality rates [80, 81]. A piRNA cluster at chromosome 17 produces piR-32051, piR-39894, and piR-43607; their over-expression significantly associates with RCC of advanced tumor stage, metastasis, and cancer-specific survival [57]. piR-57125 expression in RCC tissue is low, being lower in metastatic than non-metastatic tumors. Whereas piR-30924 and piR-38756 are associated with cancer metastasis, showing higher expression in metastatic and decreased expression in non-metastatic tumors compared to normal tissue. The higher expression of piR-30924 and piR-38756 as well as the lower expression of piR-57125 in metastatic primary tumors were significantly associated with tumor recurrence and OS [58]. Although further study of these piRNAs is needed to understand the mechanisms of novel piRNAs in KC, their different expression levels between non-metastatic and metastatic, and tumor and normal tissue suggest their potential as biomarkers for RCC diagnosis, treatment, and prognosis [57, 58].

#### Hematological malignancies

MM, the second most common hematological malignancy, is characterized by malignant plasma cell accumulation within the bone marrow. Relapse is common because it is difficult to remove all myeloma cells [82, 83]. piRNA-823 is increased in both patients with MM and cell lines, and positively linked with disease stage. piRNA-823 directly correlates with de novo DNA methyltransferases DNMT3A and 3B in primary CD138^+^ MM cells. Silencing piRNA-823 markedly reduces DNMT3A and 3B mRNA and protein, which decreases global DNA methylation and causes re-expression of methylation-silenced tumor suppressor p16_INK4A_. As VEGF secretion is also reduced, piRNA-823 modulates MM cell proliferation, apoptosis, and cell cycle progression by regulating both DNA methylation and angiogenesis [36]. Moreover, granulocytic myeloid-derived suppressor cells (G-MDSCs) enhance the stemness of MM stem cells (MMSCs) by promoting the MM cells to produce more piR-823 and DNMT3B, improving MM cell survival and maintaining their stemness [59]. Tumor-associated endothelial cells are biologically unique. They rapidly proliferate and are highly sensitive to growth factors, resistant to apoptotic stimuli, and strongly pro-angiogenic, and thereby are instrumental in tumor growth [84]. piRNA-823 accumulates in MM-derived-extracellular vesicles (EVs), which effectively transport piRNA-823 to endothelial cells, promoting their proliferation, migration, and capillary structure formation and enhancing VEGF, IL-6, ICAM-1, and CXCR4 secretion, causing their malignant transformation. MM-derived-EV-transported piRNA-823 is essential for re-educating endothelial cells toward a unique environment amenable to MM cell growth by altering their biological characteristics [60]. Thus, piR-823 is considered a promising target for MM treatment. Classical Hodgkin lymphoma (cHL) comprises 11% of all lymphomas. In cHL lymph nodes, the tumor bulk mostly comprises CD4^+^ and cytotoxic T cells, B cells, macrophages, and other cell types that crosstalk with the few “Hodgkin Reed-Sternberg” (HRS) tumor cells [85, 86]. piR-651 is highly expressed in lymph nodes of patients with cHL and associates with clinical outcome. Low piR-651 expression in HRS cells is associated with shorter disease-free survival and shorter OS, thus representing an independent prognostic factor for these measures. piR-651 can also distinguish responders vs non-responders to first line treatment [61].

#### Glioblastoma

Glioblastoma deriving from neuroepithelium is the most malignant and invasive intracranial tumor with the worst prognosis [87]. piR-30188 and PIWIL3 expression is decreased and negatively correlates with glioma pathological grade. piR-30188 suppresses tumor cell proliferation, invasion, and migration and promotes apoptosis by binding to OIP5-AS1. Low OIP5-AS1 expression increases miR-367-3p expression, thereby decreasing CEBPA, which facilitates glioma development by binding to the promoter of TRAF4 (which promotes cancer proliferation, migration, and invasion and inhibits apoptosis), ultimately weakening TRAF4 expression. Therefore, PIWIL3/piR-30188 regulates the glioma cell malignant phenotype via the OIP5-AS1/miR-367/CEBPA/TRAF4 pathway [38]. piR-8041 is also down-regulated (10.3-fold) in glioblastoma multiforme (GBM) relative to normal tissue and reduces cell proliferation by interacting with ERK1/2 mitogen-activated protein kinase (MAPK) mRNA. Up-regulated MAPK inhibits cell cycle arrest at the G 1 /S checkpoint. Furthermore, piR-8041 down-regulates several HSP and DNAJ protein family members, inhibiting cell proliferation and promoting death. piR-8041 treatment decreases glioma stem cell marker ALCAM/CD166 expression, and inhibits A172 glioma cell line but not normal human astrocyte (NHA) proliferation, suggesting the clinical value of its targeting for glioma management [62]. piRNA-DQ593109/ PIWIL1 in glioma endothelial cells increased blood-tumor barrier (BTB) permeability by binding to maternally expressed 3(MEG3) lncRNA of the MEG3/miR-330-5p/RUNX3 axis. miR-330 inhibition promoted runt-related transcription factor 3 (RUNX3) expression, which increased BTB permeability through transcriptional repression of zonula occludens 1 (ZO-1), occludin, and claudin-5 [63]. As a signaling molecule or scaffolding protein, ZO-1 recruits other signaling molecules, such as occludin, and claudin-5, which restrict hydrophilic drug absorption through the paracellular pathway [88, 89]. piRNA-DQ593109/PIWIL1 promotes therapeutic agent delivery into the glioma micro-environment, enhancing anti-tumor effects [63]. Although the features of BTB in tumor tissues differ from the blood-brain barrier (BBB), it still limits macromolecular chemotherapeutics transport into glioma tissues [90]. Finally, piR-DQ590027 is poorly expressed in glioma-conditioned ECs whereas piR-DQ590027 over-expression could decrease ZO-1, occludin, and claudin-5 expression to further increase glioma-conditioned normal BBB permeability through the piR-DQ590027/MIR17HG/miR-153(miR-377)/FOXR2 pathway. Thus, piR-DQ590027 is an attractive therapeutic target for glioma [64].

#### Other cancers

Fibrosarcoma, a soft tissue sarcoma originating from the intra- and intermuscular fibrous tissues, fascia, and tendons, is highly aggressive albeit rare. Fibrosarcomas metastasize at early stages and display genetic complexities [68]. piR-39980, a fibrosarcoma tumor suppressor, inhibits ribonucleoside-diphosphate reductase subunit M2 (RRM2) expression by binding to its 3′-UTR [65]. RRM2 subunit catalyzes the formation of dNTPs, the precursors for DNA synthesis, and regulates the anti-apoptotic protein Bcl-2 [91–93]. Therefore, two pathways underlie the function of this piRNA in fibrosarcoma oncogenesis by RRM2 targeting: RRM2 down-regulation results in failure of dNTP catalysis, leading to inhibition of cell proliferation owing to the lack of DNA synthesis; and RRM2 repression disrupts RRM2-mediated Bcl-2 regulation [65].

Ovarian cancer (OCa) is a commonly diagnosed cancer (3.4%) and cause of cancer death (4.4%) in women [68]. Among OCa types, endometrioid ovarian cancer (ENOCa) and serous ovarian cancer (SOCa) of EOCa are frequently observed and highly lethal [94]. We found that piR-52207 was up-regulated in ENOCa and piR-52207 and piR-33733 were also increased in SOCa. Up-regulated piR-52207 contains 2–21 nt binding sites with the 3′-UTR of its targets NUDT4, MTR, EIF2S3, and MPHOSPH8, which promote ENOCa cell proliferation, migration, and tumorigenesis. In SOCa, piR-33733 targets LIAS3′-UTRs, whereas piR-52207 binds ACTR10 and PLEKHA5 3′-UTRs and 5′-UTRs, leading to increased anti-apoptotic and decreased pro-apoptotic proteins. piR-52207 and piR-33733 thus participate in OCa oncogenesis through involvement in numerous cell signaling pathways at the post-transcriptional level, supporting these as possible therapeutic targets for this class of malignancy [66].

Pancreatic cancer is a highly lethal disease, for which mortality is tightly associated with incidence. Most patients with pancreatic cancer remain asymptomatic until the disease reaches an advanced stage [95]. piR-017061, located within the sno-HBII-296A snoRNA cluster, is down-regulated in pancreatic cancer, although the mechanism remains unclear [67].

### piRNAs as biomarkers in cancer

Early detection and treatment are beneficial to cancer prognosis. As piRNAs function mainly up-stream of different regulatory networks and signaling pathways, they have very high significance for early cancer diagnosis and treatment. Recently, one type of the most studied small non-coding RNAs, tumor-associated miRNAs in peripheral blood, have been described as biomarkers for cancer diagnosis [96]. RNA sequencing revealed that not only miRNAs but also other types of non-coding RNAs including piRNAs are stably present in human blood [97, 98]. piRNAs, being similar to miRNAs in length, can easily pass through the cell membrane into the circulation [99], and are extremely stable and resistant to degradation by ribonucleases in body fluid [100]. Thus, piRNAs in circulating tumor cells (CTCs) represent promising new complementary tumor markers for cancer.

As mentioned above, various piRNAs differently express between tumor tissues and matched normal tissues, associated with aggressive biological behaviors. Blood samples as a non-invasive diagnostic method, is widely used in the clinical. Here, we summarized the recent studies regarding role of piRNAs as biomarkers in blood of patients (Table 2).

**Table 2:** piRNAs as biomarkers in cancer

piRNA	Cancer	Expression in blood	Clinical correlation	ROC curve AUC sensitivity specifity	Reference
piR-651	Gastric cancer	down	TNM stage, distant metastasis	0.841 0.709 0.813	[101]
piR-823		down	TNM stage, distant metastasis	0.822 0.805 0.812	[101]
piR-5937	Colorectal cancer	down	TNM stage	0.806 0.718 0.725	[102]
piR-28876		down	TNM stage	0.8065 0.753 0.700	[102]
piR-54265		up	TNM stage, survival times and curative efficacy of chemotherapy	0.811 0.667 0.885	[45]
piR-823	Renal cell cancer	up	TNM stages	–	[103]
piR-823	Multiple myeloma	up	TNM stages	–	[60]
piR-651	classical Hodgkin lymphoma	down	the presence of lymphoma	–	[61]

piR-651 and piR-823 levels from CTCs in the peripheral blood of patients with GC are lower than those of healthy controls. Compared with the positive detection rates of serum carcinoembryonic antigen (CEA) and carbohydrate antigen 19–9 (CA19–9) levels (20.37, 31.11%, respectively), piR-651 (74.07, 71.11%) and piR-823 (88.88, 84.44%) are more sensitive, indicating that these piRNAs are more sensitive for GC screening than the commonly used biomarkers. Receiver-operating characteristic (ROC) curve analyses revealed that both peripheral blood piR-651 and piR-823 were valuable biomarkers for differentiating GC from controls with area-under-the-curve (AUC) of 0.841 and 0.822, respectively. Furthermore, their respective positive predictive values were 0.881 and0.926, with the positive likelihood ratio of piR-823 (4.301) being higher than that of piR-651 (3.785). piR-823 level was also positively associated with T stage and distant metastasis (*P* < 0.05), indicating piR-823 as a preferential biomarker for screening CTCs in GC [101].

In serum of patients with CRC, piR-5937 and piR-28876 expression decreased significantly with advanced clinical stage (*P* < 0.0005), and their diagnostic potential was high albeit for patients in clinical stage I; no correlation between piRNA expression and tumor grade, location, and size was detected (*P* > 0.05). Both piRNA levels significantly increased in serum samples of patients 1 month following surgery, suggesting that their levels are linked to the presence of the tumor. Moreover, CEA/CA19–9 are up-regulated in less than 50% of patients with colon cancer, whereas piR-5937 and piR-28876 were down-regulated in almost 70% of all tested samples. Thus, these piRNAs may serve as promising biomarkers for early colon cancer detection as well as potential novel biomarkers for patient monitoring following surgical treatment [102]. In serum, levels of a five-piRNA-panel (piR-001311, piR-004153, piR-017723, piR-017724 and piR-020365, piRNA-based Panel I) progressively decreased from those of healthy controls through patients with colorectal adenoma (CRA) to CRC. As adenoma represents a precursor stage of CRC, decreased serum piRNAs in adenomas might constitute an early indicator in cancer progression. The panel might also help identify individuals with an increased probability of developing CRC in the population with familial adenomatous polyposis. The diagnostic potential of this five-piRNA based Panel I was better than that of CEA-CA19–9 based Panel II, with sensitivity, specificity, and AUC of Panel I and Panel II being0.782, 0.750, 0.862 and 0.509, 0.9054, 0.745, respectively. Serum piR-017724 was also identified as an independent prognostic factor for CRC. Therefore, circulating piRNAs possess potential as a novel class of blood-based biomarkers for cancer [104]. Serum piR-54265, which is relatively stable in patients with CRC, positively correlates with tumor levels. piR-54265 is up-regulated in a CRC stage-dependent manner, with highest levels in patients with metastatic CRC. Specifically, the higher the serum piR-54265 level, the shorter the survival time. Furthermore, as a therapy marker, serum piR-54265 levels correlate with the curative efficacy of chemotherapy in patients with CRC. Significantly better response to chemotherapy was observed in patients with low compared to high serum piR-54265 levels (AUC of 0.811; sensitivity 66.7%, specificity88.5%) [45].

In the ROBERT’ study, we observed significantly decreased piR-823 expression in tumor tissue compared to paired non-tumorous renal parenchyma (*P* < 0.0001), whereas piR-823 levels in the serum of patients with RCC are increased compared to those of healthy controls and associated with advanced clinical stages of RCC. piR-823 levels are also significantly higher in urinary samples from patients with RCC in comparison to healthy controls. The above phenomenon might be explained by active release of piR-823 by tumor cells, leading to its decreased levels in tumors and increased levels in the circulation [103].

piRNA-823 exhibits long-term stability in EVs derived from peripheral blood. piRNA-823 is significantly increased in the peripheral EVs of patients with stage II and III MM or those with renal injury and hyphemia. Increased piRNA-823 in peripheral EVs positively correlates with higher levels of β2-MG (r = 0.800, *P* < 0.01), serum Cr (r = 0.468, P < 0.01), and lower levels of Hb (r = − 0.393, *P* < 0.05), but negatively correlates with blood calcium (r = − 0.019, *P* > 0.05) and LDH (r = 0.138, P > 0.05). Thus, piRNA-823 might serve as a potential indicator for MM prognosis and stratification [60]. piR-651 which is down-regulated in serum from patients with cHL compared to that in from healthy individuals at diagnosis, derives from circulating rather than tumor cells. The down-regulation in patients may reflect differences in the peripheral blood populations associated with the presence of lymphoma. At complete remission, levels do not markedly differ between patients and healthy controls [61].

### Piwi proteins and cancer

#### PIWIL1(HIWI)

PIWIL1, which is regulated by DNA hypomethylation, is over-expressed in lung tumor tissues, which might facilitate cancer cell proliferation, invasion, and migration and contribute to poor OS in patients with lung adenocarcinoma or malignant lung cancer phenotypes. Notably, PIWIL1may be a potential target for treatment as an epigenetic driver gene in LC [105]. PIWIL1 gene knockout using the CRISPR-Cas9 system in the AGP01 GC cell line significantly decreased in AGP01 cell migration capacity and invasiveness. PIWIL1 gene knockout results in altered expression (up- or down-regulation) of numerous genes, such as DOCK2, ZNF503, PDE4D, ABL1, and ABL2, whose encoded proteins are involved in cellular invasion and migration. Consequently, PIWIL1 may play a crucial role in the GC signaling pathway, and may be useful as a therapeutic target of GC [106]. PIWIL1 mainly localizes in the cytoplasm of CRC tumor cells. High PIWIL1 expression in tumor tissue is closely related to the tumor differentiation degree, infiltration depth, lymph vascular invasion, lymph node metastasis, and TNM stage. High PIWIL1 expression also indicates poor patient prognosis, suggesting PIWIL1 as an important molecular marker for predicting CRC prognosis [107]. Moreover, PIWIL1 genes together with piR-823 play a role in RCC pathogenesis. Decreased or absent PIWIL gene expression associates with more aggressive tumor phenotype and worse survival, indicating that PIWIL1 can serve as potential prognostic biomarkers in patients with RCC [103]. Furthermore, PIWIL1 can induce EMT and endow endometrial cancer (EC) cells with stem-like properties, such as tumor cell viability, migration, invasion, and sphere-forming activity. In addition, PIWIL1 over-expression leads to increased acquisition of CD44 and ALDH, known endometrial CSC markers. Thus, Piwil1 may become a valuable target for developing a novel treatment strategy for EC [108]. Moreover, PIWIL1 up-regulation in EC causes the loss of phosphatase and tensin homolog deleted on chromosome ten (PTEN) expression, which serves as an essential tumor suppressor role in EC through DNMT1-mediated PTEN hypermethylation [109]. Furthermore, PIWIL1 and PIWIL2 are significantly elevated in invasive ductal carcinoma (IDC), which promotes cancer development by aberrant DNA methylation resulting in genomic silencing and inducing a stem-like state of cancer cells [110] (Table 3).

**Table 3:** The role of piwi proteins in various Cancer

PIWI	Cancer	Expression	Function	Reference
PIWIL1	Lung cancer	up	DNA hypomethylation	[105]
	Gastric cancer	up	Regulate signaling pathway of gastric cancer	[106]
	Colorectal cancer	up	be used as an important molecular marker for predicting the prognosis of CRC patients	[107]
	Renal cell cancer	down	serve as potential prognostic biomarkers in patients with RCC	[103]
	Endometrial cancer	up	become a valuable target for developing a novel treatment; DNA methylation	[108, 109]
	Invasive ductal carcinoma	up	aberrant DNA methylation	[110]
PIWIL2	glioma	up	correlated with the poor prognosis	[111]
	cervical cancer	up	induced H3K9 acetylation but reduced H3K9 trimethylation,	[112]
	Non-small cell lung cancer	up	increasing the expression of CDK2 and CyclinA	[113]
	Renal cell cancer	down	connected with bad survival	[103]
PIWIL3	Glioma	down	regulate PIWIL3/piR-30,188/OIP5-AS1/miR-367-3p/CEBPA/TRAF4 pathway	[38]
	Gastric cancer	up	regulate JAK2/STAT3 signaling pathway	[114]
	Multiple myeloma	up	involve in MM progression and metastatic	[115]
PIWIL4	Triple-negative breast cancer	up	activating TGF-β, MAPK/ERK, and FGF signaling and avoiding immune recognition	[116]

#### PIWIL2(HILI)

PIWIL2 is highly expressed in glioma and correlates with poor patient prognosis. In vivo, PIWIL2 knockdown in glioma cells induces cell cycle arrest, increases apoptosis, and inhibits glioma cell migration [111]. The human papilloma virus (HPV) oncoproteins E6 and E7 can reactivate PIWIL2 during cervical cancer (CC) tumorigenesis, with Piwil2 over-expression inducing H3K9 acetylation but reducing H3K9 trimethylation, which contributes to epigenetic reprogramming and embryonic stem cell (ESC) signature maintenance. Thus, PIWIL2 plays an important role in the transformation of cervical epithelial cells to tumor-initiating cells (TICs) by epigenetic regulation [112]. PIWIL2 is up-regulated both at the RNA and protein level in malignant cancer tissues in NSCLC compared with adjacent normal tissue. It promotes cell proliferation by increasing the expression of CDK2 and cyclinA, which are essential factors that control DNA synthesis and the cell cycle. Conversely, PIWIL2 silencing results in apoptosis and G2/M cell cycle arrest [113]. In addition, low PIWIL2 expression is linked with poor survival in patients with RCC [103].

#### PIWIL3(HIWI3)

The PIWIL3/piR-30188/OIP5-AS1/miR-367-3p/CEBPA/TRAF4 pathway can regulate the biological behavior of glioma cells. PIWIL3 are expressed at low levels in glioma tissues and negatively associate with glioma pathological grade [38]. PIWIL3 over-expression promotes GC cell proliferation, migration, and invasion whereas its down-regulation suppresses the progression of GC via the JAK2/STAT3 signaling pathway [114]. PIWIL3 protein is also increased in more aggressive primary malignant melanoma and metastatic disease, and thus may be involved in malignant melanoma progression [115].

#### PIWIL4(HIWI2)

PIWIL4 is widely expressed in BC tissues and several cell lines derived from Triple-negative breast cancer (TNBC), which promotes cell survival, division, and migration of cancer by activating TGF-β, MAPK/ERK, and FGF signaling pathways, which play key roles in cancer. Moreover, PIWIL4 inhibits MHC class II expression, which may assist cancer cells to avoid immune recognition and response [116]. PIWIL2/PIWIL4 co-expression and localization in HCC may be useful as an indicator for tumor prognosis, as the transformation of negative to positive cytoplasmic PIWIL2/PIWIL4 expression indicates that tumors are in the precancerous period or in the initial stage of tumorigenesis. In comparison, transformation of negative cytoplasmic to positive nuclear expression indicates that the tumor may be more malignant. Moreover, disappearance of PIWIL2/PIWIL4 protein cytoplasmic expression leaving only nuclear expression suggested poor HCC prognosis [117].

## Conclusion

Currently, with the development of next-generation sequencing technologies and other advanced detection technologies, the different expression of piRNAs/piwi proteins can be readily detected between disease and normal stages. Because of the high morbidity and mortality of cancer, it has become a huge global health burden. Under normal circumstances, the piRNAs/piwi proteins are maintained at a stable level by the physiological balance between synthesis and degradation in germ cells and somatic cells. However, when piRNA or piwi protein expression becomes disordered, they will lose their normal functions and may result in the occurrence of cancer. In this review, we summarize several methods that are available to analyze piRNA changes in cancer (Table 4). We elaborated on the pro-cancer or anti-cancer mechanisms of some piRNA/piwi proteins in various cancers. Specifically, piRNA/piwi complexes could recruit other proteins to form pi-RISC, which degrades targeted RNA through complementary sequences, such as piR-36712 [22] and piR-DQ598677 [47]. piRNAs also recruited DNMT, causing DNA methylation at specific loci [46], and regulate the level of phosphorylation of proteins in cellular signaling pathways [44]. Several databases are available for piRNA function analyses, prediction of targeted RNAs, and searching piRNA clusters and homologous piRNAs, such as piRBase (http://www.regulatoryrna.org/database/piRNA/) and piRNABank (http://pirnabank.ibab.ac.in/). Research regarding piRNAs has mainly been focused at the transcriptional and post-transcriptional level, whereas few studies have investigated piRNA function at the post-translational level. Further research regarding the post-translational modification of piRNAs is of considerable importance for the study of tumorigenesis mechanisms. In addition, as the research on piRNA is still in its infancy, some specific functions and biosynthesis mechanisms are still under investigation. Recently, some studies have shown that many piRNAs are highly expressed in blood samples, indicating the potential for piRNAs to serve as potential tumor biomarkers, which has become a hot topic of research [104]. Compared with traditional tumor markers, piRNAs appear to be more precise and more sensitive, although their practicality has yet to tested. Moreover, piRNAs may represent therapeutic targets to inhibit the growth and division and promote the apoptosis of tumor cells via siRNA, anti-sense oligonucleotides, and CRISPR-Cas9-mediated genome editing. However, little research and applications of piRNAs in targeted therapy are available, and the mechanisms by which piRNA expression is altered in a variety of cancers has not yet been clarified. It is hoped that the advances described in this review may stimulate the additional research necessary to fully understand the basic biological mechanisms of piRNAs and their disruption, along with their potential as tools for clinical application in cancer management and treatment.

**Table 4:** The available assays of piRNAs

Common Assays	Objective of Assays	Reference
High-throughput Sequencing (HTS)	Assaying new and known piRNAs	[17, 53]
Reverse Transcription-quantitative PCR (RT-qPCR)	Assaying exact piRNA copy number per cell and the relative expression	[22]
Southern Blot	Assaying exact piRNA copy number	[53]
Nouthern Blot	Assaying the number of nucleic acids of piRNA	[22]
RNA binding protein immunoprecipitation (RIP)	Assaying the interaction of piRNA-proteins	[22, 53]
RNA Pull Down		
Luciferase reporter system	Assaying the interaction of piRNA-target RNA	[22, 65]
Microarray assay	Assaying DNA methylation by piRNAs,	[45]

## Data Availability

Not applicable.

## Abbreviations

AUC: Area under curve
BBB: Blood-brain barrier
BC: Breast cancer
BTB: Blood-tumor barrier
CA19–9: Carbohydrate antigen 19–9
CDK: Cyclin-dependent kinase
CEA: Carcinoembryonic antigen
cHL: classical Hodgkin lymphoma
CRC: Colorectal cancer
CSC: Cancer stem cells
CTCs: Circulating tumor cells
EC: Endometrial cancer
EMT: Epithelial-mesenchymal transition
ENOCa: Endometrioid ovarian cancer
GC: Gastric carcinoma
H3K9: Unmethylated histone 3 lysine 9
HCC: Hepatocellular carcinoma;
HSF1: Heat shock factor 1
HSP: Heat shock protein
KC: Kidney cancer
LC: Lung cancer
lncRNA: Long noncoding RNA
Mino: Minotaur
MM: Multiple myeloma
nt: nucleotide
OS: Overall survival
pi-RISC: piRNAs-induced silencing complex
piRNA: Piwi interacting RNA
PTEN: Phosphatase and tensin homolog deleted on chromosome ten
RCC: Renal cell cancer
SOCa: Serous ovarian cancer
TE: Transposable element
TGS: Transcriptional gene silencing
Zuc: Zucchini
